# Redox Biology of Respiratory Viral Infections

**DOI:** 10.3390/v10080392

**Published:** 2018-07-26

**Authors:** Olga A. Khomich, Sergey N. Kochetkov, Birke Bartosch, Alexander V. Ivanov

**Affiliations:** 1 Engelhardt Institute of Molecular Biology, Russian Academy of Sciences, Vavilov str, 32, 119991 Moscow, Russia; kochet@eimb.ru; 2 Inserm U1052, Cancer Research Center Lyon, University of Lyon, 69000 Lyon, France; birke.bartosch@inserm.fr; 3 DevWeCan Laboratories of Excellence Network (Labex), 69003 Lyon, France

**Keywords:** oxidative stress, reactive oxygen species, influenza virus, respiratory syncytial virus, rhinovirus, Nrf2, inflammation

## Abstract

Respiratory viruses cause infections of the upper or lower respiratory tract and they are responsible for the common cold—the most prevalent disease in the world. In many cases the common cold results in severe illness due to complications, such as fever or pneumonia. Children, old people, and immunosuppressed patients are at the highest risk and require fast diagnosis and therapeutic intervention. However, the availability and efficiencies of existing therapeutic approaches vary depending on the virus. Investigation of the pathologies that are associated with infection by respiratory viruses will be paramount for diagnosis, treatment modalities, and the development of new therapies. Changes in redox homeostasis in infected cells are one of the key events that is linked to infection with respiratory viruses and linked to inflammation and subsequent tissue damage. Our review summarizes current knowledge on changes to redox homeostasis, as induced by the different respiratory viruses.

## 1. Introduction

Redox biology embraces events involving shift of balance between reactive oxygen or nitrogen species (ROS and RNS, respectively) production and their scavenging. Changes of redox status take place during various cellular processes, including proliferation, differentiation, signaling, and metabolism [1]. Redox homeostasis also plays important roles in pathology. Accumulation of ROS or RNS or/and depletion of scavenging systems leads to the development of oxidative stress, chronic activation of immune responses, and inflammation [2]. Due to the ability of ROS to react with almost any kind of biological molecules, including proteins, lipids, and nucleic acids, their chronic elevation is generally associated with genome instability, dysfunction of organelles, and apoptosis [3].

The term “reactive oxygen species” covers short-lived oxygen-containing intermediates with high reactivity. The most extensively studied ROS include superoxide anion (O_2_^•−^), hydroxyl radical (HO^•^), and hydrogen peroxide (H_2_O_2_) [4]. The consequences of their production and corresponding antioxidant pathway, activated in response to it, depend on the location in a cell. An example of a well characterized source of O_2_^•−^ is the electron transfer to oxygen that occurs during respiration in mitochondria. A predominant ROS-scavenging enzyme that is implicated in neutralization of O_2_^•−^ in mitochondria is superoxide dismutase 2 (SOD2 or MnSOD) [5]. SOD2 converts O_2_^•−^ to H_2_O_2_, which in turn, can be metabolized by different enzymes, including catalase (CAT), peroxiredoxins (Prdx), or glutathione peroxidases (GPx) [6]. An extensive description of the various cellular ROS sources and scavenging enzymes and pathways are described further below and in the following comprehensive reviews [7,8].

Markers of redox misbalance in blood and tissues are often taken into account in the pathology of various diseases. Chronic viral hepatitis B and C are generally associated with oxidative stress, and levels of ROS and oxidized metabolites correlate with the severity of liver damage and with the risk of the development of related pathologies—fibrosis and hepatocellular carcinoma [9]. Moreover, currently redox biology pays much attention to localization of ROS sources, since local changes in ROS levels may influence signaling pathways by activating “redox switches” [10,11]. In lungs, redox homeostasis is crucial in the pathology of asthma [12]. Lung infection with respiratory viruses is, in general, associated with cytokine production, inflammation, cell death, and other pathological processes, which could be triggered by enhanced ROS production. Investigation of the influence of these infections on ROS-producing and ROS-scavenging enzymes and systems may allow for the identification of those that are crucial for replication of the pathogens and occurrence of virus-associated disease. Our review aims to summarize the known data on the role of redox biology in the pathologies that are associated with infection with respiratory viruses.

## 2. Respiratory Viruses

Respiratory infections comprise a group of diseases that affect millions of people worldwide and pose threat to many of them, especially for kids and elderly. Respiratory viruses cause infections of the upper or lower respiratory tract. The group of respiratory viruses comprises influenza (IV), human respiratory syncytial (HRSV), human rhino (HRV), human metapneumo (HMPV), parainfluenza, and adeno- and corona-viruses (severe acute respiratory syndrome coronavirus SARS-CoV). Many of them cause common clinical syndromes, including nasal congestion, cough, sore throat, and fever, although some of these pathogens may also display more specific clinical manifestations, such as bronchiolitis, pneumonia, etc. (discussed below in this section).

### 2.1. Influenza Viruses

Influenza viruses are RNA viruses belonging to the *Orthomyxoviridae* family [13]. Three IVs are infectious for humans, namely types A, B, and C. The IV genome consists of eight segments (seven for influenza C virus) encoding 9–11 proteins, depending on the type of virus. These proteins include the surface proteins hemagglutinin (HA) and neuraminidase (NA), three subunits of RNA polymerase (PA, PB1, and PB2), two non-structural proteins (NS1 and NEP), and two matrix proteins (M1 and a selective proton channel protein M2). Various subtypes of the most common influenza A viruses are classified based on the diversity in the structure of HA and NA proteins. Influenza A and B viruses cause epidemics, whereas influenza C virus is less wide spread and tends to cause infections with less severe symptoms. According to the World Health Organization (WHO), seasonal epidemics that are associated with these infections result in 3 to 5 million cases of severe illness each year, and 250 to 500 thousands deaths worldwide [14]. Currently, safe and effective vaccines are available. However, their specificity is limited to a particular subtype of the virus due to the diversity of circulating influenza A strains and ability of IV to rapidly accumulate mutations (viral antigenic drift). So, often these vaccines do not perfectly match the circulating subtype or become ineffective within short amounts of time due to viral antigenic drift, thus requiring the annual re-fabrication of vaccines and revaccination. Treatment options for IV infections are limited to inhibitors of neuraminidase, which prevent virion release and spread, but not replication [15] and blockers of the M2 proton channel [16]. However, the development of drug resistance is an important issue. Due to the high prevalence of IV serotypes that are resistant to adamantane antiviral drugs, the WHO recommends neuraminidase inhibitors instead of M2 proton channel blockers (amantadine and rimantadine) [14].

### 2.2. Human Respiratory Syncytial Virus

Human respiratory syncytial virus belongs to the *Pneumoviridae* family of RNA viruses [17]. The viral genome is represented by a single-stranded negative-sense 15.2 kb RNA encoding 11 proteins. Similarly to IV, HRSVs are classified as A or B genotypes, according to the structure of fusion (F) and attachment (G) proteins. The rest of the viral proteome includes proteins N, M, P, SH, L (polymerase), M2-1, M2-2, and NS1, NS2. HRSV is a leading cause of bronchiolitis and pneumonia in children, especially in pre-mature infants and children with cardiac or pulmonary diseases. It was also shown that HRSV significance in adults is comparable to nonpandemic IV [18]. The group of risk includes the elderly and immunocompromised people. Approximately 33 million cases of infection are reported annually worldwide [19,20]. No HRSV vaccine is available yet. The only prophylactic treatment option, palivizumab (Synagis^®^)—a monoclonal antibody against the F viral protein is recommended for HRSV prevention and the treatment of infants at high risk of severe disease and complications. Other therapeutic approaches, including ribavirin administration, are discussed in detail elsewhere [21].

### 2.3. Human Rhinovirus

Human rhinovirus, the major and most prevalent cause of the common cold, belongs to the *Picornaviridae* family of RNA viruses [13]. The HRV genomic single-stranded positive-sense 7.2 kb RNA encodes 11 proteins. Viral capsid is formed by proteins VP1–4; non-structural proteins include polymerase three-dimensional (3D), two proteases 2A and 3C, proteins 2B, 2C, 3A, and 3B. HRV replicates at 33–35 °C, thus it mostly infects nose epithelial cells. Therefore, direct HRV-induced pathologies manifest as nasal congestion, rhinitis, and sore throat. However, sometimes rhinoviral infection leads to more severe disease, including bronchiolitis, pneumonia, and exacerbations of chronic pulmonary disease. Therapy of infection is mostly supportive [22]. Several antivirals with preventive mode of action (e.g., capsid binding for abrogation of viral RNA release into cells) were in clinical trials, including pleconaril, vapendavir, and rupintrivir, however, to the best of our knowledge, none of them were approved and recommended for HRV therapy. Supportive treatment may include *Echinacea*, vitamine C, and zinc.

Parainfluenza virus belongs to the *Paramyxoviridae* family, with its genome being represented by a single-stranded RNA. Parainfluenza virus infects both upper and lower respiratory tracts and usually causes common cold-like symptoms. The virus is known to be dangerous for young children; however, it also infects adults. Sendai virus (SeV) known as former murine parainfluenza virus belongs to the same *Paramyxoviridae* family. SeV is negative sense, single-stranded RNA virus, which is capable of infecting rodents and other animals. It is accepted as a model of respiratory infection. Adenoviruses are members of *Adenoviridae* family and have DNA genome. Adenovirus infections are usually mild, and only symptomatic treatment may be required.

## 3. Enhanced ROS Production during Viral Respiratory Infections

Many lines of evidence suggest that marked signs of increased production of ROS accompany all respiratory viral infections. However, none of the published data are based on direct measurement of ROS levels using electron paramagnetic resonance technique. In all existing papers levels of ROS were assessed indirectly either by using redox-sensitive dyes (2′,7′-dichlorodihydrofluorescein diacetate, DCFHDA; dihydroethidium, DHE, etc.) that are oxidized by ROS into quantifiable fluorescent products or via quantification of cellular oxidation products. In patients that are infected with IV marked increases in DNA (8-hydroxydeoxyguanosine), lipid (malondialdehyde, F2-isoprostane, 7-ketocholesterol, and 7β-hydroxycholesterol), and protein (carbonyl content) oxidation products in blood plasma and urine were reported [23,24,25]. Elevated levels of sterol oxidation products were detected not only during the infection but also three months after IV clearance [24]. Increased levels of ROS as well as of nitric oxide synthase 2 (iNOS) and nitrotyrosine content representing markers of nitrosative stress were also reported in lung tissues of patients that died in the fatal IV pandemics [26]. IV-infected mice and cell lines also exhibit an enhanced production of ROS and disturbance of antioxidant defense [27,28,29,30] and are, therefore relevant models of IV infection for investigation of the changes in redox homeostasis induced by the virus.

Augmented ROS production is not only a feature of IV. Other respiratory viruses also promote ROS production. Elevation of total ROS levels in A549 airway cells is also triggered by HRSV [31,32] and in various cell lines by SeV [33,34], as was shown with DCFHDA. Consistently, the accumulation of lipid peroxidation products and oxidized glutathione (GSH) were reported in plasma of infants with HRSV-induced acute bronchiolitis [35]. SeV, as shown above for IV, also triggers the induction of iNOS, enhanced production of nitric oxide, and accumulation of nitroguanosine [36]. A reduced antioxidant capacity, another marker of oxidative stress, was detected in HRSV-infected infants, mice, and cells [37,38,39,40]. Reduced levels of antioxidant enzymes were also reported in airway cells and in mice replicating human HMPV [37,41]. HRV was shown to induce production of ROS in airway cells by enhancing O_2_^•−^ production and by depleting intracellular GSH [42,43,44].

## 4. Sources of ROS in the Infected Cells

**Figure 1:**
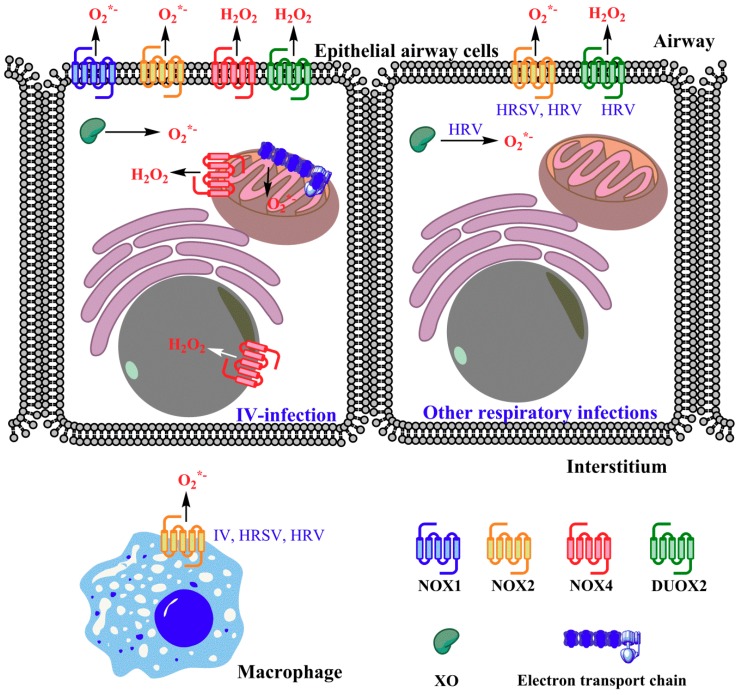
Sources of reactive oxygen species (ROS) in airway epithelial cells infected with influenza virus (IV, the left cell) or human respiratory syncytial virus (HRSV) and rhinovirus (HRV, the right cell). Cellular events, triggered by ROS production, are detailed in the text. The sources are mainly represented by nicotinamide adenine dinucleotide phosphate oxidases (NADPH oxidases, Nox), Dual oxidase (Duox) and xanthine oxidase (XO). Respiratory viruses also contribute to the production of superoxide anion by Nox2, an enzyme expressed in macrophages and, to a lesser extent, in epithelial cells.

Respiratory viruses are known to induce ROS-generating enzymes, including nicotinamide adenine dinucleotide phosphate oxidases (NADPH oxidases, Nox) and xanthine oxidase (XO) and to disturb antioxidant defenses. Increased activities of the Nox and Dual oxidase (Duox) family were observed both in vitro and in vivo. Treatment of infected cell cultures and laboratory animals (mice) with the pan-Nox inhibitor dibenziodolium chloride (DPI) profoundly attenuated ROS production induced by IV [29], HRSV [45,46], and HRV [44]. Although DPI has low specificity for Nox in comparison to other flavoproteins [47], a detailed analysis with other approaches revealed that several NADPH oxidases are implicated in generation of ROS: Nox1, Nox2, Nox4, and Duox2 (Figure 1).

Nox2 is a phagocytic enzyme that contributes to virus-induced ROS production during in vitro and in vivo infection with IV [30,48,49,50], HRSV [50,51], HRV [44,50], and SeV [50,51]. In Nox2^−/−^ knockout mice, the IV-induced ROS and RNS increase was less pronounced [48]. Moreover, virus titer and virus-induced inflammation were also lower in these mice, and were correlated with improved lung functions [52]. It is generally acknowledged that Nox2 activation requires phosphorylation of its regulatory subunit p47^phox^/NCF1 (neutrophil cytosolic factor 1) [53]. In line with this, Nox-derived ROS production during infection with inactivated H5N1 IV is attenuated in NCF^−/−^ mice when compared to wild type littermates, and this effect is accompanied by improved lung functions [54]. Similarly, HRV infection in cells lacking NCF1 is accompanied by significantly lower levels of O_2_^•−^ and H_2_O_2_ [44]. Noteworthy that Nox2 is expressed in macrophages, monocytes, neutrophils, and, to a lesser extent, in epithelial cells [53,55,56], see also a review of Grandvaux et al. [57] for detailed analysis of expression of Nox isoforms in different respiratory tract sections and cell lines. To et al. recently reported that respiratory RNA viruses induce Nox2-mediated ROS production in endosomes of alveolar macrophages [50]. In addition, recruitment of neutrophils and monocytes from the bloodstream to the infection site may contribute significantly to the enhanced production of superoxide anions by Nox2 during the infection. Indeed, influenza [58,59] and HRSV [60] cause a pronounced migration of neutrophils to the respiratory tract. However, to the best of our knowledge, input of macrophages or neutrophils into Nox2-derived production of O_2_^•−^ has not been assessed for any of these viruses.

Another NADPH oxidase, Nox4, was also shown to be a source of ROS in lung cancer cells or in primary airway epithelial cells infected with IV [29,61]. Amatore et al. described induction of Nox4 and reduction of Nox2 in in vitro IV infections. However, in the context of other respiratory virus infections, the activity of Nox4 has not been studied. Interesting data exist on the possible involvement of Nox1 in the regulation of ROS production in IV-infected mice [62]. O_2_^•−^ production in BALF (bronchoalveolar wash fluid) cells of Nox1 knockout mice infected with IV subtype H3N2 is similar to that of wild-type littermates during the early stages of infection (day 3), but suppressed at late stages of infection (day 7). This suggests that Nox1 plays no or only a marginal role in the production of O_2_^•−^. Nox1 knockout furthermore significantly augments production of proinflammatory cytokines and chemokines and overall inflammation in mice at three days post-infection with no effect on viral titer, but it suppresses cytokine production at later stages of disease when the viral titer significantly decreases, indicating the resolution of the infection. These findings suggest a protective role of Nox1 during IV infection, which is not limited to changes in total ROS production [62]. However, recent work from another group pointed to a role for Nox1 in the induction of ROS. Survival of IV-infected mice with inactive Nox1 was prolonged [63], suggesting that Nox1 is a source of ROS that is hazardous. In HRV-infected polarized airway epithelial cells Nox1-derived ROS are responsible for triggering barrier dysfunction, a dangerous process leading to decreased transepithelial resistance, thus making the host susceptible to bacterial pathogens [64,65]. Interestingly, ROS generation and disruption of the barrier during HRV infection also depend on one of the double-stranded RNA (dsRNA) receptors, Nod-like receptor X-1 (NLRX-1) [65]. This receptor has mitochondrial location and is able to activate nuclear factor kappa B (NFκB) via ROS [66]. However, direct mechanisms underlying this effect remain unknown. In the case of HRSV, down-regulation of Nox2, but not of Nox1 by RNA interference affects NFκB signaling [51], even though NFκB is one of the ROS-dependent transcription factors [67] responsible for cytokine and chemokine gene expression. Thus, involvement of Nox1 in direct induction of oxidative stress during respiratory viral infection remains controversial. Another source of ROS during IV infection that belongs to the Nox/Duox family is Duox2 [61,68,69]. IV triggers, both in vitro and in vivo, a pronounced induction of Duox2 and DuoxA2 and a moderate down-regulation of Duox1 [69,70]. Noteworthy, Duox2/DuoxA2 induction is induced by H1N1 but not by the H3N2 subtype [69]. Duox2-mediated production of ROS is required in the redox regulation of type 1 and 3 interferon (IFN) antiviral response pathways in airway cells (see Section 6 below).

Similar data also exist for other viruses. Fink et al. described the elevation of the transcripts encoding Duox2, Nox4, and a regulatory subunit of Nox2-p67^phos^ in cells infected with SeV and HRSV [51]. This paper also shows absence of notable changes in transcription of other NADPH oxidases. They did not assess contribution of Nox/Duox to ROS production during SeV or HRSV infection. Instead, they showed that two pan-Nox inhibitors (DPI and apocynin) and knockdown of Nox2 but not of Nox1 or Nox5 prevented activation of the NFκB pathway by these viruses. However, in their subsequent paper, it was shown that SeV does induce Duox2 at protein level and increases extracellular levels of H_2_O_2_, whereas HRSV almost does not [57]. In the case of SeV, the mechanism of induction involves production and secretion of two cytokines: IFNβ and tumor necrosis factor alpha (TNFα), the accumulation of which activates Duox2 gene transcription through signal transducer and activator of transcription protein/interferon regulatory factor 9 (Stat2/IRF9)-dependent Stat1 independent pathway. In contrast, HRSV blocks this pathway and thus does not allow for the induction of Duox2.

HRV-infected human primary tracheobronchial epithelial cells also trigger induction of Duox2 [71]. Patients with chronic obstructive pulmonary disease (COPD) have increased the susceptibility to HRV and HRV-infected airway epithelial cells from COPD patients show elevated levels of Duox1 and Duox2 [72].

Xanthine oxidase is another ROS-generating enzyme that is induced by IV [73,74]. XO catalyzes the conversion of hypoxanthine to xanthine and further to uric acid thus being implicated in the catabolism of purine nucleic bases [75]. Elevation of XO levels was observed in serum, lung tissues, and in bronchoalveolar wash fluid of IV-infected mice [73,74]. Effective inhibition of O_2_^•−^ production with allopurinol, a classical XO inhibitor, validated the significant contribution of this enzyme to virus-induced ROS production [73]. Up-regulation of XO was also shown for HRV [43], although Kaul et al. reported the absence of the inhibitory effect of allopurinol towards HRV-induced ROS production and subsequent interleukin 8 (IL8) induction [44]. Whether XO contributes to HRSV-mediated enhancement of ROS generation is unknown.

Finally, mitochondria are known to be an important source of O_2_^•−^ in IV infection due to the virus-induced leakage of electrons from the respiratory chain [61,76]. However, this is unlikely due to a direct interaction of viral proteins with the electron transport chain, indeed data supporting mitochondrial localization of viral proteins have not been reported so far. Enhanced production of ROS in mitochondria may rather be mediated by Duox2 [61] or Nox2 (reviewed in [77,78]), both of which have been shown to induce mitochondrial dysfunction. So far, there is no data concerning the disruption of the mitochondrial electron transport by other respiratory viruses, and this area of research clearly requires further studies. For SeV there is indirect evidence that this virus does not affect production of ROS in mitochondria: activation of caspase 9, a key event in SeV-induced apoptosis, is insensitive to ROS scavengers and is not accompanied by cytochrome c release, a frequent consequence of severe mitochondrial dysfunction [79].

Finally, another interesting mechanism of O_2_^•−^ production was shown for IV and SeV [36]. It involves the induction of iNOS, production of nitric oxide, formation of nitroguanosine, and the latter together with iNOS and P450 reductases leads to the production of this type of ROS. Indeed, the same group described a mechanism of O_2_^•−^ formation that involves single-electron reduction of a 8-nitro-guanosine into the respective anion-radical with NADPH-cytochrome P450 reductase (P450 reductase), any isoform of NO-synthase (NOS) or even xanthine oxidase, and the subsequent transfer of the electron to molecular oxygen [80]. Interestingly, such data are missing for any other virus that triggers oxidative stress.

Investigations of the molecular mechanisms by which respiratory viruses induce massive ROS production and in the particular identification of the viral proteins that are responsible for these effects are scarce in comparison to viruses, such as human immunodeficiency virus (HIV) and hepatitis B and C viruses (HBV and HCV, respectively) [9,81]. Amongst the few reports covering this topic are reports on the IV M2 [82] and PB1-F2 [83] proteins. Overexpression of M2, a proton channel protein, activates protein kinase C (PKC) and increases the production of ROS that was detected using DCFH2DA and MitoSOX as readouts [82]. PB1-F2 decreases SOD1 expression resulting in impaired ROS scavenging [83]. These data are corroborated by a study of Flory et al. who pointed at M, HA, and NP proteins as activators of NFκB via IκB kinase (IKKβ), whose effects can be suppressed by antioxidants [84]. In respect to HRSV infection, one single paper suggests that the generation of ROS is augmented by the viral fusion (F) protein [45]. Treatment with DPI abrogates ROS production in F-expressing cells suggesting the involvement of NADPH oxidase(s). However, since DPI also inhibits xanthine oxidase, proteins of mitochondrial electron transport chain, and other flavin-dependent enzymes [47], the role of NADPH-oxidases remain to be explored. Finally, the overexpression of SARS-CoV 3CL-Pro (protease) protein induced the generation of ROS and NFκB activation; however, the molecular mechanisms are unknown [85].

## 5. Respiratory Viruses and Antioxidant Defense Pathways

Respiratory viruses not only enhance ROS production but also affect cellular defense systems against ROS. Despite the important role of physiological levels of ROS in signaling, chronically elevated levels cause severe oxidative injury. The antioxidant defense system is comprised of a variety of enzymes and transcription factors, as well as an array of low molecular weight molecules that are often referred to as antioxidants. These latter compounds directly scavenge ROS, participate in recycling of the defense enzymes, or regulate redox-sensitive transcription factors.

The key transcription factor that controls expression of an array of defense enzymes is nuclear factor E2-related factor 2 (Nrf2) [86]. During normal levels of ROS generation, Nrf2 is retained in the cytoplasm by kelch-like ECH-associated protein 1 (Keap1), which targets Nrf2 to ubiquitin-dependent degradation. When ROS production is enhanced, Nrf2 dissociates from Keap1 and translocates into the nucleus, where it binds the antioxidant response element (ARE) in the promoter regions of target genes encoding predominantly antioxidant enzymes. Noteworthy, the Nrf2 gene itself also contains ARE-like sequences in its promoter [87], thus amplifying its expression in a positive feedback loop. Amongst the Nrf2-regulated target genes are superoxide dismutases, catalase, peroxiredoxins, and glutathione peroxidases. O_2_^•−^ are converted into H_2_O_2_ by three SOD isoforms in mammalian cells: cytoplasmic SOD1, mitochondrial SOD2, and extracellular SOD3 (EC-SOD). Soluble peroxides are eliminated by catalase, peroxiredoxins, and glutathione peroxidases. The peroxiredoxin family comprises six different members with redox-active cysteine residues. Peroxiredoxins differ by localization and mechanism of re-activation. The eight members of the GPx family display different localization patterns and activities in the cell and they have varying affinities for different types of peroxides. Noteworthy, GPx4 and Prdx6 are capable of neutralizing lipid peroxides in addition to soluble peroxides [88,89]. Finally, many antioxidant defense enzymes are involved in the biosynthesis of natural antioxidants, such as glutathione and the maintenance of a reduced pool of GSH. These include glutathione synthetase, reductase and peroxidase, thioredoxin reductase, and others.

IV strains are thought to activate the Nrf2/ARE defense pathway in vitro and in mice by inducing nuclear translocation and transcriptional activity of Nrf2 because transcription of the Nrf2 target gene heme oxygenase 1 (HO-1) was shown to be augmented [90,91]. Interestingly, differences between seasonal and especially pandemic H1N1 subtypes versus highly pathogenic H7N9 and H5N1 strains in their capacities to promote Nrf2 phosphorylation and the subsequent translocation/activation have been reported [92]. Jacoby et al. did not find any changes to SOD1 levels in IAV-infected cells [93], however, other groups observed a decrease of SOD1 as mediated by proteasomal degradation of specificity protein 1 (Sp1), a transcription factor that drives the expression of the *SOD1* gene [94,95]. An increase in SOD1 expression was found in patients with asymptomatic influenza A infection [96]. Decline in total SOD activity was reported in children infected with H1N1 subtype [25]. So, SOD1 status during IV infection is still questionable.

Similar controversy exists over other antioxidant enzymes, such as CAT and indoleamine 2,3-dioxygenase (IDO). The latter scavenges O_2_^•−^ and uses them for oxidation or conversion of tryptophan into kinurenin [97]. Levels of IDO were shown to be unaltered and levels of catalase decreased in IAV-infected cells in vitro, [93], whereas in infected mice, IDO and HO-1 were induced and CAT levels remained unchanged [98]. Yamada et al. reported depletion of CAT and Prdx6 in mice infected with subtype H1N1, however, this was due to depletion of the IV permissive bronchial Clara and/or alveolar type 2 (AT2) cells in which these enzymes are predominantly expressed [91]. In addition, the same study also reported the pronounced induction of several other antioxidant enzymes, namely, GPx3 and HO-1.

Unlike IV, HRSV reduces mRNA levels and levels of Nrf2 in nuclei of airway epithelial cells [38]. Indeed, HRSV is capable of inducing Nrf2 deacetylation and subsequent proteasomal degradation, which leads to the down-regulation of expression of antioxidant enzymes [99]. However, the residual Nrf2/ARE pathway activity does play an important role in protection against HRSV-induced ROS production: Nrf2^−/−^ infected mice show more severe oxidative stress and inflammation when compared to their wild-type littermates [100]. Moreover, Nrf2 deficiency leads to the activation of the NFκB pathway and subsequent cytokine production. This Nrf2-NFκB crosstalk may play pivotal roles in in the associated pathogenesis, and there is clear lack of information about it for respiratory viruses. HRSV exhibits different effects on the antioxidant defense system depending on the duration of the infection. A transient activation has been reported during the first several hours post infection in lung carcinoma cells in vitro, as exemplified by induction of SOD1, SOD2, GST (glutathione S-transferase), catalase, and GPx. With progression of disease in cell culture, only SOD2 remained up-regulated resulting in enhanced H_2_O_2_ production, whereas other antioxidant enzymes, including those that are responsible for neutralization of H_2_O_2_, were suppressed [38,101]. In mice and children, no signs of induction of antioxidant enzymes were reported; instead, their levels fell down during infection [37]. HRSV infection also induced the accumulation of oxidized forms of several members of the Prdx family, including cytoplasmic Prdx1, mitochondrial Prdx3, and endoplasmic reticulum-residing Prdx4, without changes to their total protein levels [102]. This indicates that massive ROS production occurs in the respective organelles of the infected cells, since peroxiredoxins scavenge peroxides in their close proximity. Moreover, down-regulation of Prdx1 and 4 by RNA interference (RNAi) led to a more pronounced increase of ROS levels [102].

Information on the role of individual viral proteins in changes to the redox status and redox-associated processes is very scarce and is based on one article, which reports an NS1-mediated, Nrf2-independent suppression of SOD2 [103]. In this paper, HRSV NS1 protein prevented the activation of the JAK/STAT1 pathway (Janus kinases/Signal Transducer and Activator of Transcription proteins) and decreased expression of genes bearing GAS and ISG promoter sequences (interferon-gamma activated sequence and interferon stimulated genes, respectively), such as SOD2 [103]. However, *SOD2* gene expression is normally induced by NFκB rather than by IFNγ signaling [104]. Therefore, the above-mentioned effect might be explained by possible alterations of the NFκB pathway by the viral NS1 protein.

Few studies have investigated the antioxidant defense system of the host cell in human HMPV and HRV infections. HMPV has been shown to increase levels of SOD2 but to down-regulate SOD3, catalase, glutathione S-transferases, and peroxiredoxins 1, 3, and 6 [41]. In contrast, HRV does not affect activity of SOD2, catalase, and GPx, but increase levels and activity of SOD1 [105].

For SeV, we did not find any literature on interference of the virus with expression of antioxidant defense enzymes. However, a fluctuating reduction of the total GSH content has been reported in SeV-infected cells [106]. Noteworthy, this decrease was observed during the first hour post-infection and then again 24 h post-infection. Initial reduction of GSH content was due to damage of the host cell membrane during virus penetration, whereas the secondary reduction resulted from massive incorporation of cysteine into the viral proteome. However, in neither case, any changes in levels of GSSG were observed.

In addition to Nrf2, there are other transcription factors that belong to this family: Nrf1 and Nrf3. To date very little is known about functions of Nrf3 in the absence of infection. Functions of Nrf1 are defined. Nrf1 can also bind to ARE in the promoters of antioxidant defense genes, though to a lesser extent than Nrf2 (reviewed in [107]). Nrf2 and Nrf1 knockout animals have distinct phenotype meaning that functions of these two factors do not fully overlap. But again, there are no data about status of Nrf1 during respiratory as well as other viruses. So, the investigation of their effect on Nrf1 and study of a role of the latter in viral replication and pathology seems to be a nice direction for future studies.

## 6. Role of ROS in the Life Cycle and Propagation of Respiratory Viruses

So far, the evidence for a direct impact of ROS on the life cycles of respiratory viruses is very scarce. ROS-induced cell death and lysis can favor release and spread of virions and thus stimulate the replication of those respiratory viruses with a lytic life cycle. At the same time, ROS are known to contribute to suppression of some respiratory infections through the induction of innate immune responses. On the other hand, antioxidant therapies are known to ameliorate and improve disease outcome.

Overexpression of SOD1 or treatment with antioxidants reduce intracellular levels of IV polymerase thus providing a possible mechanism of the viral titer reduction in response to antioxidant treatment [94]. The observation that content of GSH negatively correlates with IV replication in infected cells or mice further corroborates this finding [108,109]. Similar results were obtained upon the inhibition of additional ROS-producing enzymes or Nox2 knockout [29,48]. However, ROS were also shown to have antiviral effect but due to modulation of immune responses.

Activation of immune responses during IV infection is achieved partly through Duox2-mediated induction of viral dsRNA sensors RIG-I and MDA5 (retinoic acid-inducible gene I and melanoma differentiation-associated protein 5, respectively) [68,70], pattern recognition receptors that trigger type I IFN responses. IV also enhances production of interferons λ1 (IL29), and λ2/3 (IL28A/IL28B), again via ROS [61]. ROS scavenging or suppression of ROS production by Duox2 or mitochondria leads to the inhibition of IFNλ synthesis and secretion, and thus the enhancement of viral replication. Induction of type I and III interferons is likely to be achieved through the Nrf2 pathway, since activation of the latter in response to IV infection or chemical inducers (i.e., sulforaphane and epigallocatechin gallate) is accompanied by an increased expression of antiviral mediators RIG-I, IFNβ, and MxA [110]. Nrf2 overexpression negatively affects the replication of IV, whereas knockdown leads to enhanced virus entry and replication [90,110]. However, this effect is unlikely to be mediated through interferon-stimulated genes, such as *MX1* and *OAS1* (2′,5′-oligoadenylate synthetase 1), since their levels are not altered during Nrf2 overexpression in the absence of the infection and are decreased in infected cells [90].

One of the mechanisms by which IV interferes with the interferon pathway is via the activation of the ROS-sensitive transcriptional factor NFκB, which controls proinflammatory cytokine production. Indeed, in IV-infected cells SOCS3 (suppressor of cytokine signaling 3) is induced through the activation of NFκB [111], whereas prevention of NFκB activation leads to the reduction of viral titers, but cytokine production also decreased [112]. Another proviral role of NFκB is the inhibition of β- and γ-catenin-dependent transcription of interferon-stimulated genes [113]. However, NFκB can also exhibit antiviral properties by activating interferon β gene transcription. Viral protein NS1 is able to prevent NFκB activation by interacting with IKK, which is required for NFκB signaling [114]. Overall, the balance between pro- and anti-viral activities of NFκB remains to be analyzed.

An interesting piece of evidence came from an investigation of SeV and its effects on the expression of Duox2 [115]. It showed that the replication of this virus is controlled by Duox2 and by the H_2_O_2_ Duox2 produces. Induction of Duox2 in SeV-infected cells is achieved through a combined action of IFNβ and TNFα that are synthesized and secreted from the infected cells through IRF3 and NFκB, respectively. In turn, Duox2 contributes to secretion of type I and III interferons. However, it should be mentioned that the detailed mechanism underlying the antiviral effect of Duox2 remains vague, and several possible assumptions have been made (see [57]).

HRSV is known to evade the antiviral effect of Duox2. Fink et al. showed that HRSV triggers the production and secretion of functional IFNβ and TNFα to a level comparable to that known to induce Duox2 in SeV infection [115]. However, HRSV blocks the Stat2/IRF9 pathway and thus controls Duox2 gene expression. Other groups found that activation of IFNs by HRSV is achieved through enhanced ROS production and the consequent STAT signaling [116]. Among the various ROS-sources, NADPH oxidases are the major triggers of STAT signaling, since treatment with Nox inhibitors abrogates STAT and RANTES (regulated on activation, normal T cell expressed, and secreted) induction [116]. Specifically, HRSV- and SeV-induced activation of NFκB and IRF3 signaling and downstream proinflammatory and antiviral signaling is initiated by sensing viral RNA by Rig-I and MDA5 helicase receptors with subsequent involvement of mitochondrial antiviral-signaling protein (MAVS) and TNF receptor-associated factor 6 (TRAF6) adaptors [56,117,118]. All of these processes depend in Nox2 [117,118], which controls the expression of MAVS [56]. Not much is known about the mechanisms of viral interference with the above mentioned pathway, however nonstructural viral proteins NS1 and NS2 are thought to be implied, albeit in an opposing way—these proteins inhibit IFN, IRF-3, and NFκB activation [119,120,121,122,123]. Thus, the virus antagonizes IFN signaling.

The role of ROS in the life cycle of HRV remains mostly unexplored. However, ROS have been reported to stimulate expression of intercellular adhesion molecule-1 (ICAM1), a major receptor for entry of this virus [124]. In line with this, treatment with reduced GSH decreased virus-mediated ICAM1 activation.

Finally, an interesting piece of evidence comes from investigation of SeV. Ciriolo et al. showed that this virus causes a pronounced decrease in GSH content in infected cells [106]. Supplementation with exogenous GSH inhibits the replication of the virus [125], whereas depletion, as caused by buthionine sulfoximine (BSO) that interferes with biosynthesis of this tripeptide, in contrast—increases virus titers [106]. However, the exact mechanism by which GSH content is linked to virus replication remains unknown. It definitely does not involve modulation of mitochondrial status, as mitochondria-derived ROS do not affect SeV propagation [126]. Finally, such investigations are missing for other respiratory viral infections.

## 7. ROS in Respiratory Virus Pathology

Alterations of ROS-producing and scavenging pathways that are caused by respiratory viral infections are implicated in inflammation, lung epithelial disruption, and tissue damage, and, in some cases, even pulmonary fibrosis. These events are at least partially interregulated: inflammation can contribute to lung damage and epithelial dysfunction and vice versa. While there is a clear correlation between markers of oxidative stress and severity of the disease in chronic hepatitis B and C [9,127,128,129], for respiratory viruses, such clinical data are scarce. The observation that ROS are implicated in the pathology of these viruses is mainly based on experimental infection models. Death of lung epithelial cells that are infected with IV in vitro occurs through enhanced ROS production, whereas antioxidants prevent cell death and also reduce damage in lungs of IV-infected mice [90,130,131,132]. GPx1 was shown to play a particular role in the pathogenicity of IV infection. In mice lacking H_2_O_2_-scavenging enzyme, GPx1, IV infection results in a more severe and longer BALF inflammation as compared to their wild type littermates [133]. Moreover, these animals exhibit higher levels of proinflammatory tumor necrosis factor alpha (TNFα), macrophage inflammatory proteins MIP-1a and MIP-2 expression in lung and elevated number of IV-specific CD8^+^ T cells in spleen. Such decrease of GPx1 can also result from selenium deficiency. Selenium deficiency leads to down-regulation of GPx1 and catalase and increased production of mucosa and altered epithelial morphology [134]. However, there are no direct data showing that IV does interfere with selenium metabolism and expression of the key Se-metabolizing proteins.

Similar data that point to an important role of ROS in the associated pathology also exist for SARS-CoV [85,135] and HRSV [32]. DNA damage and a proliferation arrest with consequent activation of cellular senescence program was observed in HRSV-infected immortalized epithelial cells and in the respiratory epithelia of HRSV-infected mice even after viral clearance [32]. Overexpression of catalase, treatment with *N*-acetylcysteine (NAC) or with cell-permeable reduced glutathione ethyl ester (GSHee) reversed these effects, strongly implying the accumulation of ROS in these events [32]. However, contradictory data also exist. For example, Olsen et al. demonstrated that influenza A and B-induced apoptosis of Madin-Darby Canine Kidney (MDCK) cells cannot be blocked by antioxidants or compounds protecting mitochondria [136]; moreover, the cell permeable calcium chelator BAPTA-AM severely augmented cell death, whereas ionomycin, a calcium ionophore, blocked apoptosis. For SeV the data are contradictory: Gao et al. reported that virus-induced apoptosis can be prevented by treatment with NAC [33], whereas Bitzer et al. found that the activation of caspases in SeV-infected cells is not sensitive to antioxidants [79].

One of the key events of inflammation is the interaction of airway epithelial and endothelial cells with leukocytes. It is mediated by a set of cell adhesion molecules, such as VCAM1 (vascular cell adhesion protein 1), ICAM1, and E-selectin, which trigger and augment infiltration with leukocytes [137,138]. At least for HRV it was demonstrated that infection leads to induction of ICAM1. ICAM1 induction was successfully blocked by addition of reduced GSH [124] showing that ROS play a particular role in endothelial cell mediated leukocyte recruitment.

It is well known that IV, HRSV, and other viral infections in vivo trigger massive production of proinflammatory cytokines and chemokines, such as TNFα, IL6, and IL8 [139,140], referred to as cytokine storm and thus trigger cell death [141]. The cytokine storm is responsible for lung tissue damage during viral respiratory infections. Activation of immune cells is also necessary for infection elimination, but it should be taken into account that in many cases not viruses themselves, but associated host immune response is more fatal for tissue integrity and functionality. One of the key mediators of induction of cytokines and chemokines is NFκB. NFκB signaling is directly activated by ROS or/and by certain proinflammatory cytokines, such as TNFα and IL1β, and drives in turn expression of a wide spectrum of other cytokines and chemokines [142]. These include interleukins 2, 6m and 8, MIP1a, MCP-1 (monocyte chemoattractant protein 1), and RANTES [142]. Consequently, NFκB is a key player that coordinates initiation of innate and inflammatory responses as well as maturation of lymphocytes within the adaptive immune system [142]. Respiratory viruses induce NFκB signaling both in vitro and in vivo in a ROS-dependent fashion [42,51,54,84,139]. Several IV proteins, including M, HA, and NP activate NFκB [84]. In IV-infected mice, NFκB activation is accompanied by the increased production of IL6 and IL8, TNFα, CCL5/RANTES, CXCL10 (C-X-C motif chemokine 10) [139]. Treatment with NAC abolishes NFκB activation and cytokine production in infected cells [143]. However, contrary data also exist: Mastronarde et al. noticed that although induction of IL8 by RSV is sensitive to antioxidants such as NAC, the latter inhibits binding of AP-1 and NF-IL6 factors to the promoter region of *IL8* gene, but it does not affect the binding of NFκB [144]. So, a precise action of ROS in induction of proinflammatory cytokines merits further studies.

Mechanisms of cytokine induction by HRV infection are less clear. On the one hand, low molecular weight antioxidants including NAC and reduced GSH abolish both NFκB activation and cytokine production during HRV-infection, thus pointing to ROS as key mediators of both effects [42]. Moreover, XO has a significant input in NFκB, since its inhibitor allopurinol effectively prevents the activation of this transcription factor in a cell culture model [43]. Several other studies pointed to Nox2-generated ROS as triggers of NFκB activation and concomitant production of proinflammatory cytokines, including TNFα and RANTES [51,117]. However, Kim et al. reported that HRV infection can induce IL6 and IL8 in an NFκB-independent manner [145]. Distinct mechanisms seem to activate NFκB during HRV infection, and they imply NLRX-1-mediated ROS generation [65,66]. Not much is known about HMPV-mediated cytokine induction. Although HMPV activates NFκB, just like other respiratory viruses, its SH protein, when being expressed by itself, is capable of preventing this activation [146]. Disturbance of the redox balance in HRSV-infected airway epithelial cells such as A549 cells, induces IL8 [140], MCP-1, and interferon regulatory factor 3 (IRF-3). The latter binds the promoter of the chemokine RANTES [31,147,148]. RANTES activates different types of immune cells in lung inflammatory infiltrates during HRSV infection. It was recently shown that other proteins of the inflammatory response, namely cytokine High Mobility Group Box 1 (HMGB1), were also upregulated by HRSV infection, in an at least partially ROS-dependent manner [149]. Another possible mechanism of HRSV-associated ROS production and proinflammatory cytokines is activation of immune responsive gene 1 (IRG1), since its inhibition/suppression during infection abrogates immune cell infiltration and reduces ROS production and lung injury in infected mice [150]. This enzyme catalyzes production of itaconic acid from *cis*-aconitate, an intermediate of tricarbonic acid (TCA) cycle [151]. It is well described that IRG1 induction leads both to enhanced ROS production presumably through promotion of the pentose phosphate pathway [152] and through controlling succinate levels [153] as well as to activation of the Nrf2 pathway through direct alkylation of the Nrf2 partner—Keap1 [154]. A recent paper also showed that IRG1 can be induced by type I interferons [154]. NFκB pathway activation during HRSV infection is at least partly mediated by the ROS-dependent activation of MSK1 (mitogen- and stress-activated protein kinase 1), one of the kinases responsible for NFκB serine phosphorylation [39]. Also, as described above, phosphorylation of NFκB p65 Ser536 requires the induction of Nox2 that triggers MAVS expression, thus allowing for RigI- and MDA5-mediated sensing of viral RNA and concomitant TRAF6-IKKβ-NFκB signaling [117] (see Section 6 above).

Production of cytokines is controlled not only transcriptionally, but also by proteolytic cleavage of precursor polypeptides by cellular complexes, referred to as inflammasomes into active cytokines [155,156]. Inflammasomes contain sensor molecules that are connected to caspase 1 via a specialized adaptor protein. A majority of complexes contain NOD-like receptor (NLR) sensor molecules, among which the best characterized is NLRP3. Induction of this sensor is not sufficient for the activation of NLRP3 inflammasomes. To date, three possible mechanisms of NLRP3 inflammasome activation have been proposed. One of them involves mitogen-activated protein kinase-mediated ROS signaling [157]. Respiratory (IV, HRSV) as well as non-respiratory (encephalomyocarditis, measles) viruses all activate NLRP3 inflammasomes via ROS [46,158]. Mechanistically, ROS production by NADPH oxidases has been implied [46], however mitochondrial ROS were also shown to play a role [159]. Data on HRV- and SARS-CoV-mediated NLRP3 inflammasome activation is scarce and it just shows that disturbance of calcium homeostasis plays a role [160,161]. The endoplasmic reticulum (ER) is the main storage of calcium ions in a cell. Calcium signaling can be induced in many cases by events referred to as ER overload response (EOR) that occurs during accumulation of some viral and several host cell proteins in the ER [162,163]. One of the major consequences of EOR is an activation of the mitogen-activated protein kinase MAPK/NFκB cascade, which plays crucial roles in inflammation regulation [164,165]. So far, IV has been shown to induce ER stress and concomitant classical unfolded protein response (UPR) [166]. At the same time, the induction of EOR in infected cells and its role in the activation of inflammasomes remains to be elucidated.

**Figure 2:**
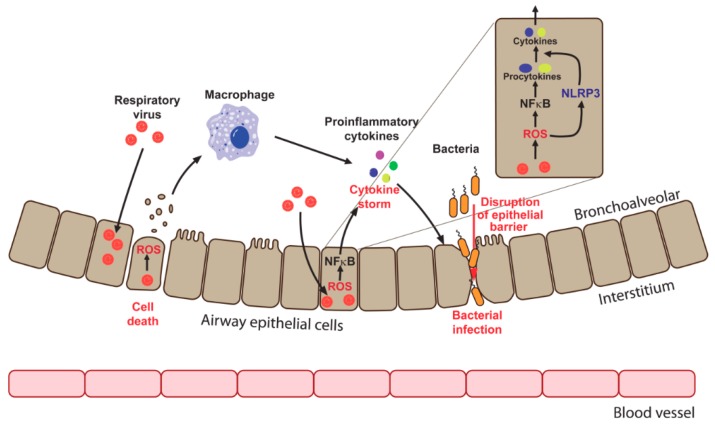
Mechanisms of cytokine production (cytokine storm) and epithelial barrier disruption by respiratory viruses. Infection leads to the enhanced ROS production that may trigger cell death and subsequent macrophage activation. This activation is accompanied by cytokine production leading to the inflammation and destruction of epithelial cell contacts. Proinflammatory cytokines could also be produced by infected cells via activation of redox-sensitive nuclear factor kappa B (NFκB) pathway that drives transcription of their genes and via activation of NLRP3 inflammasome in ROS-dependent manner that mediates maturation and secretion of cytokines. Disruption of epithelial barrier results in the increased susceptibility to bacterial infection.

Another pathological consequence of enhanced production of ROS in the presence of respiratory viruses is a barrier dysfunction, which leads to an increased susceptibility of the host to bacterial pathogens (Figure 2). HRV e.g., disrupts airway epithelial barrier function with a consequent increase of barrier leakage and susceptibility to pathogens [64,65,167]. Suppression of Nox1 using pharmacological inhibitors or small interfering RNAs (siRNAs) prevented barrier disruption, revealing the role of this oxidase in the development of HRV-associated tissue damage [64]. HRSV also deteriorates barrier function through a mechanism involving the activation of protein kinase D (PKD) [168]. Although a causative role of enhanced generation of ROS was not investigated in that study, the fact that PKD is redox regulated is well known [169,170]. Interestingly, discrepant data were reported for IV infection. IV-infected differentiated primary porcine airway epithelial cells maintained barrier functions despite the loss of ciliated cells [171]. However, in other publications, IV was shown to disrupt cellular tight junctions both in vitro and in infected mice [172,173]. Recently, a connection between IV-induced Nox2-mediated ROS production and enhanced sensibility to post-IV *Staphylococcus aureus* pneumonia was revealed [174]. Concomitant treatment of co-infected mice with both antibiotics and a Nox2 inhibitor increased the survival of animals. Bacterial pneumonia is the most dangerous consequence for IV patients, and this work may help to improve treatment strategies. However, the authors did not investigate the link between the observed sensitivity to bacterial infection and the disruption of epithelial resistance. So, the clinical importance of epithelial barrier dysfunction in the settings of IV infection remains to be elucidated.

## 8. Antioxidant Therapy of Respiratory Viruses

Despite the antiviral role of cytokines, their “over” production during the cytokine storm is more fatal for lung tissues in respiratory infections than the viruses themselves [175]. Therefore, various agents have been evaluated and they are used as remedies to target not only the viruses but also the virus-associated inflammation. Much attention has been paid to antioxidants due to the correlation between severity of lung injury and the markers of oxidative stress in lung and blood of patients that are infected with HRSV [35,37] or IV [24,54]. Here, we briefly summarize the major findings for treatment of respiratory infections with antioxidants. More detailed data can be found in the following reviews [101,176,177,178].

The most studied agents targeting ROS that were evaluated for treatment of IV and HRSV infections are listed in Table 1. Noteworthy, almost no data exist regarding efficacy of antioxidant therapies in humans. Instead, the most studied antioxidants—NAC, ascorbic acid and vitamin E were shown to have positive effects such as decrease of virus replication and inflammation in cells and mice infected with IV and/or HRSV.

It should be noted that there is no strong link between HRSV viral titer and the severity of disease during antioxidant treatment. For example, treatment with an inhibitor of NO production ameliorates inflammation during HRSV infection, but it increases viral replication both in cell culture and in a mouse model [179,180]. Another interesting approach for antioxidant treatment proposed recently is usage of hydrogen sulfide donors [181]. It is based on the fact that HRSV infection is accompanied by depletion of H_2_S due to decreased expression of H_2_S-generating enzyme cystathionine γ-liase (CSE) [182]. Since H_2_S and its donors are able to prevent mitochondrial dysfunction, oxidative stress, and reduce inflammation, this fact provides another piece of evidence that respiratory viruses interfere with cellular redox-sensitive pathways.

An additional strategy is inhibition of ROS-generating enzymes such as Nox2 in the case of IV. Detailed analysis of such an approach is presented in several excellent reviews [183,184]. Briefly, the most studied inhibitors, gp91dstat and apocynin, inhibit the association of Nox2 with p47^phox^ and prevent the assembly of the active complex of the oxidase. Both of the inhibitors were shown to reduce inflammation in IV-infected mice thus validating perspectiveness of antioxidant therapy of IV [48,50].

Data on the treatment of HRV with antioxidants are scarce and they have only been produced in in vitro systems. Pyrrolidine dithiocarbamate (PDTC), an antioxidant and inhibitor of NFκB, inhibits HRV replication and the processing of the viral polyprotein in cells [211,212]. Other well studied antioxidants, such as ascorbic acid, Trolox, 2-mercaptoethanol, and NAC, do not display any promising therapeutic activity [211]. Carbocisteine and ambroxol reduce HRV titers at least partly via down-regulation of the viral receptor ICAM1 [213,214].

## 9. Conclusions and Future Perspectives

Respiratory viruses cause millions of cases of severe illness and thousands deaths each year. So far, no efficient measures for prevention and treatment exist. Future research in these areas will be vital. These infections are associated with pronounced inflammation. Importantly, the virus-induced ROS production and disturbance of the host’s redox balance are important triggers of inflammation. Oxidative stress is triggered in many distinct ways, including the induction of ROS-generating enzymes and disturbance of antioxidant defense. However, not enough is known about mechanisms of virus-associated oxidative stress and the subsequent consequences for cells, tissue, and the organism. Many conflicting data on the antioxidant defense status and role of ROS in viral propagation exist and need to be resolved based on in vitro as well as clinical studies. In particular, techniques that allow for localizing ROS production and scavenging within the cell, and to trace exact oxidative reactions of ROS with host cell proteins will be required to resolve these questions.

Production of ROS can induce cell death and the release of virions representing possible proviral role of enhanced ROS production and altered redox balance. On the other hand, one of the important roles of oxidative stress is the triggering of an antiviral immune response. However, too strong immune responses lead to a cytokine storm and severe inflammation, which is very dangerous for tissue and may disturb lung function. From this point of view, antioxidant supplementation is expected to ameliorate the consequences of infection. Many studies showed the positive role of antioxidant therapy in infected cells and animals. At the same time, almost no relevant clinical data exist even for popular antioxidants, such as NAC, ascorbic acid, and vitamin E. This gap needs to be filled in by new research.

## Figures and Tables

**Table 1:** Antiviral activity of antioxidants and antioxidant enzymes towards respiratory viral infections.

Antioxidant	Influenza Virus				Human Respiratory Syncytial Virus			
	Model System	Antiviral Activity	Synergism with	Ref	Model System	Antiviral Activity	Synergism with	Ref
*N*-acetylcysteine (NAC)	Cells Mice Human	Yes	Ribavirin, oseltamivir	[185,186,187,188]	Cells	? *	NA	[31,32,143,189]
Pyrrolidine dithiocarbamate (PDTC)	Cells Mice	Yes	ND **	[132,190]	ND	ND	ND	ND
Glutathione	Cells Mice	Yes	ND	[108,109]	Cells	Yes	ND	[32]
Butylated hydroxyanisole (BHA)	-	-	-	-	Cells Mice	No	ND	[31,191]
SOD1	Cells Mice	No	Rimantadine	[74,95,192]	Rats	Yes	ND	[193]
SOD2	Mice	No	Ribavirin	[194]	Rats	Yes	ND	[193]
SOD3	Mice	No	ND	[195]	ND	ND	ND	ND
Roflumilast	-	-	-	-	Cells	Yes	NA	[196]
Carbocisteine	Cells	Yes	ND	[197]	Cells	Yes	NA	[198]
Ascorbic acid	Cells Mice	Yes	ND	[199,200,201]	ND	ND	ND	ND
Ambroxol	Mice	Yes	ND	[202]	ND	ND	ND	ND
Resveratrol	Cells Mice	Yes	ND	[203]	Cells Mice	Yes	ND	[204,205]
Vitamin E or Trolox	Cells Mice	? ***	ND	[206,207,208]	ND	ND	ND	ND
Catalase	Mice	Yes	ND	[209,210]	Cells	No	ND	[32]

* No antiviral activity [31]; antiviral activity was reported [32,143,189]. ** No data. *** Not studies, ND [206,207]; antiviral activity was reported [208].
